# Knowledge, attitudes, and practices among guardians toward inherited retinal diseases: a structural equation modeling analysis

**DOI:** 10.1038/s41598-026-54188-7

**Published:** 2026-05-21

**Authors:** Xiaomei Chen, Yanfang Ran, Nianlian Wen, Sifu Fan, Min Wen, Zirun Feng, Jiali Jiang, Sheng Huang

**Affiliations:** 1 Zunyi Medical University, Zunyi, Guizhou, 563000 China; 2 Department of Ophthalmology, TongRen City People’s Hospital, Tongren, 554300 Guizhou China; 3 Jishou University Medical College, Jishou, 416000 Hunan China

**Keywords:** Retinal diseases, Guardians, Knowledge, Attitude, Practice, Cross-sectional studies, Questionnaires, Diseases, Health care, Medical research

## Abstract

This study aimed to investigate the knowledge, attitudes, and practices (KAP) among guardians toward inherited retinal diseases (IRDs) in children. This multicenter cross-sectional study, conducted from June 23, 2024, to February 23, 2025, invited guardians of children diagnosed with IRDs at the outpatient or inpatient departments of Tongren City People’s Hospital and its affiliated hospitals to complete a questionnaire. Path analysis was employed to examine the relationships among KAP. This study included 459 participants (51.2% male; mean age 51.44 ± 8.12 years). The KAP scores for KAP were 8.32 ± 1.61 (range: 0–17), 33.25 ± 3.51 (range: 10–50), and 36.93 ± 3.76 (range: 10–50), respectively. Path analysis revealed that knowledge had a significant direct effect on attitudes (β = 0.22, *P* = 0.023), and attitudes had a significant direct effect on practices (β = 0.66, *P* < 0.001). Although the direct effect of knowledge on practices was not significant, an indirect effect was observed through attitudes (β = 0.15, *P* = 0.024). Guardians of children with IRDs have limited knowledge and relatively negative attitudes, yet their practices are comparatively positive. These findings highlight the need for enhanced disease-related education on clinical variability to improve parental knowledge, foster more positive attitudes, and promote proactive health practice.

**Supplementary Information:**

The online version contains supplementary material available at10.1038/s41598-026-54188-7.

## Introduction

Inherited retinal diseases (IRDs) are a heterogeneous group of genetically determined retinal disorders characterized by progressive photoreceptor degeneration and visual impairment. The prevalence of IRDs, particularly retinitis pigmentosa (RP), has been estimated at approximately 1 in 3,000–7,000 in the general population, with studies in Chinese cohorts suggesting similar rates close to 1 in 3,500-1 in 3784, underscoring the public health importance of these conditions in China^1,2^. IRDs are a group of clinically and genetically heterogeneous disorders characterized by photoreceptor degeneration or dysfunction, often leading to progressive and severe vision loss across various age groups^3,4^. IRDs are a significant cause of childhood blindness, imposing substantial physical, psychological, and economic burdens on patients and their families^5^. As many IRDs manifest early in life, children are particularly vulnerable to long-term disability, making parental involvement a cornerstone of disease management. Guardians’ understanding and decision-making not only influence the timing of diagnosis and intervention but also affect emotional adjustment, treatment adherence, and long-term quality of life^6^. As primary caregivers and key decision-makers, guardians directly impact the clinical outcomes and management strategies for children with IRDs. Despite significant advances in genetic testing and counseling, guardians’ knowledge, attitudes, and practice (KAP) regarding these conditions remain inconsistent^7^. Some guardians, influenced by cultural beliefs, exhibit limited understanding or reluctance toward genetic testing, while others actively seek available medical resources^8^.

The KAP framework is a widely adopted structured approach in public health research, premised on the assumption that knowledge acquisition shapes attitudes, which in turn influence practice^9,10^. It is frequently employed to evaluate population responses to health issues and to guide targeted interventions. Parental KAP significantly affects treatment adherence and family adaptation, yet research on the KAP of guardians of children with IRDs remains limited. Existing literature highlights that, in China, guardians of children with IRDs face substantial challenges, including limited access to genetic counseling, misconceptions about the disease, and persistent cultural stigma^8^. While some KAP studies have been conducted in the context of other pediatric conditions, comprehensive assessments specific to IRDs are lacking. Given the critical role of KAP study in public health for elucidating the drivers of practice through the evaluation of knowledge, attitudes, and practice^11^, this study aims to systematically assess the KAP status of Chinese guardians of children with IRDs, providing evidence-based insights for subsequent disease education and interventions.

## Methods

### Study design and participants

This multicenter cross-sectional study was conducted from June 23, 2024, to February 23, 2025, across the following participating centers: Department of Ophthalmology, Tongren City People’s Hospital (primary center); Department of Ophthalmology, Qianjiang Central Hospital; Department of Ophthalmology, Jishou University School of Medicine; Xiaogan Aier Eye Hospital; Department of Ophthalmology, Tongren Traditional Chinese Medicine Hospital; Department of Ophthalmology, Shiqian County People’s Hospital; Department of Ophthalmology, Dejiang County People’s Hospital; Department of Ophthalmology, Sinan County People’s Hospital; and Department of Ophthalmology, Jiangkou County People’s Hospital. Guardians of children diagnosed with IRDs from the outpatient and inpatient departments of Tongren City People’s Hospital and several affiliated hospitals across China were invited to complete a questionnaire. The study was approved by the Ethics Committee of Tongren City People’s Hospital (Approval No.: 202476). Informed consent was obtained from all participants prior to data collection.

Participants were included if they met all of the following criteria: (1) being the legal guardian (parent or primary caregiver responsible for daily care and medical decisions) of a child diagnosed with an IRD before the age of 18.; (2) the participant (guardian) must be an adult aged ≥ 18 years; (3) the child was receiving care at an outpatient or inpatient department of a participating hospital during the study period; and (4) the participant (guardian) was able to understand the Chinese questionnaire and provide voluntary written informed consent. Exclusion criteria were: (1) patients or guardians unable to comprehend or complete the questionnaire due to cognitive impairment, illiteracy, severe mental illness, or language barriers; (2) refusal to sign the informed consent form; (3) non‑primary caregivers when multiple guardians were present; (4) questionnaires identified during quality control as having a completion time < 90 s, containing logical inconsistencies between Attitude Question 1 (A1) and Attitude Question 8 (A8) (same question), or having incomplete data; and (5) legal guardian of a non- IRDs child.

Notably, when a single child was accompanied by multiple caregivers during an outpatient or inpatient visit, only one primary guardian was invited to participate in the survey. The primary guardian was defined as the individual most involved in the child’s daily care, medical decision-making, and hospital visits, or the person who first provided informed consent.

### Classification system for visual impairment

All enrolled patients were assessed for visual impairment using the 2019 World Health Organization (WHO) classification system, as specified in the 11th revision of the International Classification of Diseases (ICD-11), which categorizes vision impairment as mild, moderate, or severe (Table 1)^12^.

**Table 1:** Classification system of vision impairment.

Classification of vision impairment	Variables	Distance visual acuity worse than	Distance visual acuity equal to or better than
Mild	Snellen, metres	6/12	6/18
	Snellen, feet	20/40	20/60
	LogMAR	0.30	0.50
	Decimal	5/10 (0.5)	0.3
Moderate	Snellen, metres	6/18	6/60
	Snellen, feet	20/60	20/200
	LogMAR	0.5	1.00
	Decimal	3/10 (0.3)	0.1
Severe	Snellen, metres	6/60	3/60
	Snellen, feet	20/200	20/400
	LogMAR	1.00	1.30
	Decimal	1/10 (0.1)	0.05

### Questionnaire design

A structured, self-developed questionnaire was utilized to collect data in this study. The development process was informed by existing guidelines and relevant literature^3,13,14^. During the initial development stage, the draft questionnaire was refined with input from three experts (two senior ophthalmology professors and one genetic disease specialist) until basic consensus on its content was achieved. Throughout this process, the experts evaluated each item for relevance, importance, clarity, and comprehensibility, as well as the overall face validity of the instrument. Furthermore, prior to implementing the survey on a broader scale, a pilot test involving 49 participants was conducted to assess the instrument’s reliability and comprehensibility. All returned questionnaires were complete and included in the analysis, and the internal consistency of the overall scale was demonstrated to be high, with a Cronbach’s alpha coefficient of 0.925.

The final questionnaire comprised 47 items across four dimensions. The knowledge dimension consisted of 14 items, encompassing true/false, single-choice, and multiple-choice questions. True/false and single-choice questions were scored as 1 point for correct answers and 0 points for incorrect or “not sure” responses. Multiple-choice questions were awarded 2 points for selecting all correct options, 1 point for partial correctness, and 0 points for incorrect answers, with a total score range of 0 to 17. The attitude dimension included 10 items, assessed using a five-point Likert scale from “Strongly agree” (5 points) to “Strongly disagree” (1 point), with a total score range of 10 to 50. The practice dimension comprised 10 items, also evaluated on a five-point Likert scale from “Very consistent” (5 points) to “Very inconsistent” (1 point), with a total score range of 10 to 50.

### Outcome measures

Outcome measures for this study were derived from the KAP framework. Participants achieving scores exceeding 80% of the maximum possible value in each respective domain were categorized as having adequate knowledge, a positive attitude, or sufficient practice^15^.

### Data collection and quality control

The questionnaire was administered using both electronic and paper formats. For the electronic version, the Wenjuanxing platform (https://www.wjx.cn) was used to design and distribute the questionnaire via a WeChat-accessible QR code. For the paper version, trained research assistants provided on-site clarification.

To ensure data quality, electronic questionnaires were restricted to one submission per IP address, with all questions set as mandatory. Paper questionnaires were reviewed on-site by research assistants for completeness and accuracy. During data cleaning, strict criteria were applied to exclude logically inconsistent or incomplete responses. This standardized approach ensures the reliability and validity of the dataset.

### Sample size

The minimum required sample size for this cross-sectional study was calculated using a single population proportion formula:

$$\:\mathrm{n}={\left(\frac{{\mathrm{Z}}_{1-\frac{{\upalpha\:}}{2}}}{{\updelta\:}}\right)}^{2}\times\:\mathrm{p}\times\:(1-\mathrm{p})$$

Assuming a 95% confidence level (Z = 1.96), a margin of error of 5% (d = 0.05), and an estimated proportion (p) of 0.5 to maximize sample variability, the minimum sample size was determined to be 384 participants. To account for potential non-response and invalid questionnaires, an additional 25% was added, yielding a target sample size of approximately 480 participants.

### Statistical analysis

Continuous variables were expressed as mean ± standard deviation (mean ± SD) and compared using one-way analysis of variance (ANOVA) or the Mann-Whitney U test. Categorical variables are summarized as frequencies and percentages. Spearman’s rank correlation analysis is used to evaluate correlations between KAP scores. A two-step approach was used for the structural equation modeling. First, Confirmatory Factor Analysis (CFA) was conducted to validate the factorial structure of the KAP questionnaire. The model demonstrated an acceptable fit, as indicated by the following indices: chi-square to degrees of freedom ratio (CMIN/DF) < 5.0, Root Mean Square Error of Approximation (RMSEA) < 0.08, Comparative Fit Index (CFI) > 0.8, Tucker-Lewis index (TLI) > 0.8, and Incremental Fit Index (IFI) > 0.8 (Table S1). Second, after confirming the measurement model, a path analysis was conducted to examine the mediating role of attitudes between knowledge and practices, estimating the following hypotheses: (1) knowledge had direct impacts on attitude; (2) knowledge had direct impacts on practice; (3) attitude had direct direct impacts on practice; 4) knowledge had indirect impacts on practice. We clarify that the present study employed a saturated path analysis (df = 0) using only observed variables. In such models, global fit indices (e.g., χ²/df, CFI, RMSEA) are uninformative because the model exactly reproduces the observed covariance matrix, resulting in perfect fit statistics. Consequently, these indices are neither reported nor interpreted in saturated path analyses. In addition to the path analysis, we performed bootstrapping with 1,000 bootstrap samples to estimate the 95% confidence intervals for the indirect effects of knowledge on practices through attitudes. This method was used to provide a more reliable assessment of the indirect effects and account for sampling variability. All statistical analyses are conducted using STATA versions 18.0 (StataCorp LLC, College Station, TX, USA). A two-sided p-value < 0.05 is considered statistically significant.

## Results

### Participant selection

A total of 500 responses were initially collected. After applying quality control measures—including exclusion of responses with completion time under 90 s (*n* = 1), inconsistent or abnormal IRD diagnosis responses (*n* = 1), reports of normal vision (*n* = 1), respondents under 18 years of age (*n* = 14), and logical inconsistencies between A1 and A8 (*n* = 24)—a final dataset of 459 valid responses was retained for analysis.

All questionnaires were included in the final analysis, yielding a valid response rate of 91.8%. The internal consistency of the overall questionnaire was high, with a Cronbach’s alpha coefficient of 0.925.

### Demographic information

Among 459 participants (51.2% male; mean age 51.44 ± 8.12 years), predominantly parents (61.22%). Most had a college or bachelor’s degree (53.81%) and resided in urban areas (52.29%). According to parental reports, the most common diagnosis among children was retinitis pigmentosa (61.44%), predominantly affecting males (59.26%), with a mean diagnosis age of 16.86 ± 11.62 years. Over half of the children (57.95%) had moderate visual impairment. KAP scores were 8.32 ± 1.61 (range: 0–17), 33.25 ± 3.51 (range: 10–50), and 36.93 ± 3.76 (range: 10–50), respectively. Caregivers with higher education (college or above) had significantly better attitude (33.63 ± 3.60 vs. 32.79 ± 3.34, *P* = 0.019) and practice scores (37.35 ± 3.92 vs. 36.43 ± 3.48, *P* = 0.028) than those with lower education. Urban residents scored higher in attitude (33.59 ± 3.50 vs. 32.87 ± 3.48, *P* = 0.025) and practice (37.35 ± 3.92 vs. 36.46 ± 3.51, *P* = 0.030) than rural residents. Higher annual income was associated with improved attitude (33.73 ± 3.72 vs. 32.69 ± 3.16, *P* = 0.002) and practice scores (37.67 ± 3.95 vs. 36.08 ± 3.32, *P* < 0.001).

Caregivers with family members affected by IRDs demonstrated significantly higher scores in both attitude (35.42 ± 3.61 vs. 32.75 ± 3.29, *P* < 0.001) and practice (39.60 ± 3.75 vs. 36.32 ± 3.48, *P* < 0.001) compared to those without such family history. Similarly, those who had received education related to IRDs exhibited significantly higher scores in attitude (35.54 ± 3.90 vs. 33.10 ± 3.44, *P* = 0.011) and practice (38.15 ± 3.92 vs. 36.79 ± 3.72, *P* = 0.013) than those without such education. Furthermore, caregivers of children with mild visual impairment achieved significantly higher attitude (34.25 ± 3.40, *P* < 0.001) and practice scores (38.24 res in att < 0.001) compared to other groups. The demographic characteristics of the participants are summarized in Table 2.

**Table 2:** Demographic characteristics and KAP scores.

*N* = 459	*N* (%)	Knowledge score		Attitude score		Practice score	
		Mean ± SD	*P*	Mean ± SD	*P*	Mean ± SD	*P*
Total score		8.32 ± 1.61		33.25 ± 3.51		36.93 ± 3.76	
Patient’s gender			0.281		0.173		0.071
Male	272(59.26)	8.37 ± 1.47		33.06 ± 3.48		36.65 ± 3.62	
Female	187(40.74)	8.25 ± 1.79		33.50 ± 3.53		37.32 ± 3.92	
Type of IRDs			0.128		0.204		0.446
Retinitis Pigmentosa (RP)	282(61.44)	8.29 ± 1.66		33.24 ± 3.53		36.95 ± 3.60	
Leber Congenital Amaurosis (LCA)	61(13.29)	8.04 ± 1.44		32.91 ± 3.17		36.67 ± 3.43	
Stargardt Disease	35(7.63)	8.31 ± 1.72		33.57 ± 3.44		37 ± 4	
Choroideremia	42(9.15)	8.54 ± 1.51		32.57 ± 3.32		36.16 ± 4.20	
Other	39(8.5)	8.71 ± 1.43		34.20 ± 4.00		37.92 ± 4.47	
Age of patient was diagnosed with IRDs	16.86 **±** 11.62						
Current vision status			0.244		0.002		0.001
Mild vision loss	91(19.83)	8.47 ± 1.64		34.25 ± 3.40		38.24 ± 3.83	
Moderate vision loss	266(57.95)	8.24 ± 1.57		33.07 ± 3.43		36.59 ± 3.50	
Severe vision loss	102(22.22)	8.39 ± 1.67		32.81 ± 3.67		36.61 ± 4.07	
Gender			0.212		0.667		0.973
Male	235(51.2)	8.38 ± 1.52		33.35 ± 3.45		36.93 ± 3.76	
Female	224(48.8)	8.25 ± 1.69		33.13 ± 3.57		36.91 ± 3.75	
Age (years)	51.44 **±** 8.12						
Relationship to the patient			0.387		0.001		0.001
Father	141(30.72)	8.30 ± 1.59		32.70 ± 2.99		36.27 ± 3.22	
Mother	140(30.5)	8.15 ± 1.43		32.71 ± 3.27		36.48 ± 3.37	
Other	178(38.78)	8.46 ± 1.74		34.10 ± 3.89		37.79 ± 4.25	
Residence			0.197		0.025		0.030
Urban	240(52.29)	8.4 ± 1.59		33.59 ± 3.50		37.35 ± 3.92	
Rural	219(47.71)	8.23 ± 1.62		32.87 ± 3.48		36.46 ± 3.51	
Annual household income, CNY			0.332		0.002		0.0001
≤ 100,000	215(46.84)	8.26 ± 1.60		32.69 ± 3.16		36.08 ± 3.32	
> 100,000	244(53.16)	8.37 ± 1.61		33.73 ± 3.72		37.67 ± 3.95	
Education			0.923		0.019		0.028
Senior high school/Technical secondary school and below	212(46.19)	8.28 ± 1.50		32.79 ± 3.34		36.43 ± 3.48	
Associate/Bachelor’s degree and above	247(53.81)	8.35 ± 1.69		33.63 ± 3.60		37.35 ± 3.92	
Marital status			0.066		0.323		0.915
Other (unmarried/divorced/widowed)	41(8.93)	7.95 ± 1.90		32.90 ± 4.08		37.24 ± 4.92	
Married	418(91.07)	8.35 ± 1.57		33.28 ± 3.45		36.89 ± 3.62	
Aware of IRDs			0.772		0.281		0.316
Yes	418(91.07)	8.33 ± 1.63		33.31 ± 3.47		36.96 ± 3.73	
No	41(8.93)	8.14 ± 1.37		32.60 ± 3.85		36.58 ± 3.98	
Education related to IRDs			0.783		0.011		0.013
Yes	48(10.46)	8.44 ± 2.12		35.54 ± 3.90		38.15 ± 3.92	
No	411(89.54)	8.31 ± 1.54		33.10 ± 3.44		36.79 ± 3.72	
Family member with IRDs			0.448		0.0001		< 0.001
Yes	85(18.52)	8.42 ± 1.56		35.42 ± 3.61		39.6 ± 3.75	
No	374(81.48)	8.29 ± 1.62		32.75 ± 3.29		36.32 ± 3.48	
Diagnosed with a IRDs			0.427		0.001		0.010
Yes	63(13.73)	8.46 ± 1.57		34.79 ± 3.94		38.20 ± 4.47	
No	396(86.27)	8.30 ± 1.61		33.00 ± 3.37		36.72 ± 3.59	
Pathogenic gene for IRDs			0.085		0.007		0.028
Yes	36(7.84)	8.72 ± 1.68		33.72 ± 3.36		36.55 ± 3.27	
No	13(2.83)	9.46 ± 2.87		36.61 ± 4.13		40 ± 4.54	
Not sure	410(89.32)	8.25 ± 1.53		33.1 ± 3.45		36.86 ± 3.73	

### Distribution of responses to knowledge, attitude, and practice

**Fig. 1:**
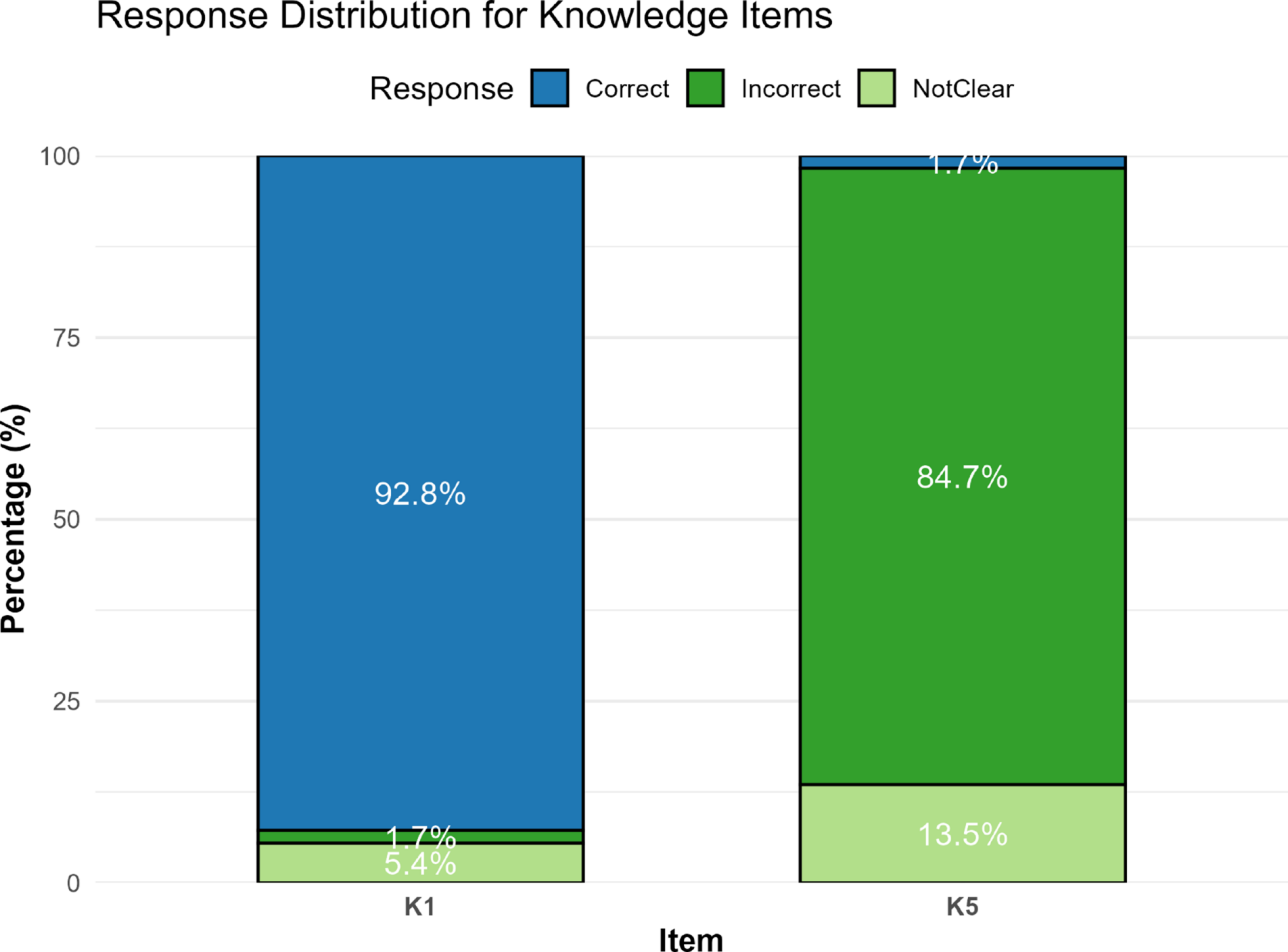
Key questions analysis of knowledge.

Knowledge dimension: The highest correct response rates were observed for the questions “IRDs may be passed down through genes” (92.81%), “Patients with IRDs need to undergo genetic testing” (84.53%), and “IRDs can be prevented” (84.75%), indicating general awareness of heredity and the importance of testing. In contrast, the lowest correct or confident response rates were found in understanding clinical variability (only 17.65% correctly recognized that the same gene can lead to diverse presentations), knowledge of treatment applicability (70.59% were unsure whether drug therapy, gene therapy, and supportive care apply), and recognition of mitochondrial inheritance (only 12.2% identified this correctly), suggesting notable gaps in more nuanced genetic understanding (Table S2 and Fig. 1).

**Fig. 2:**
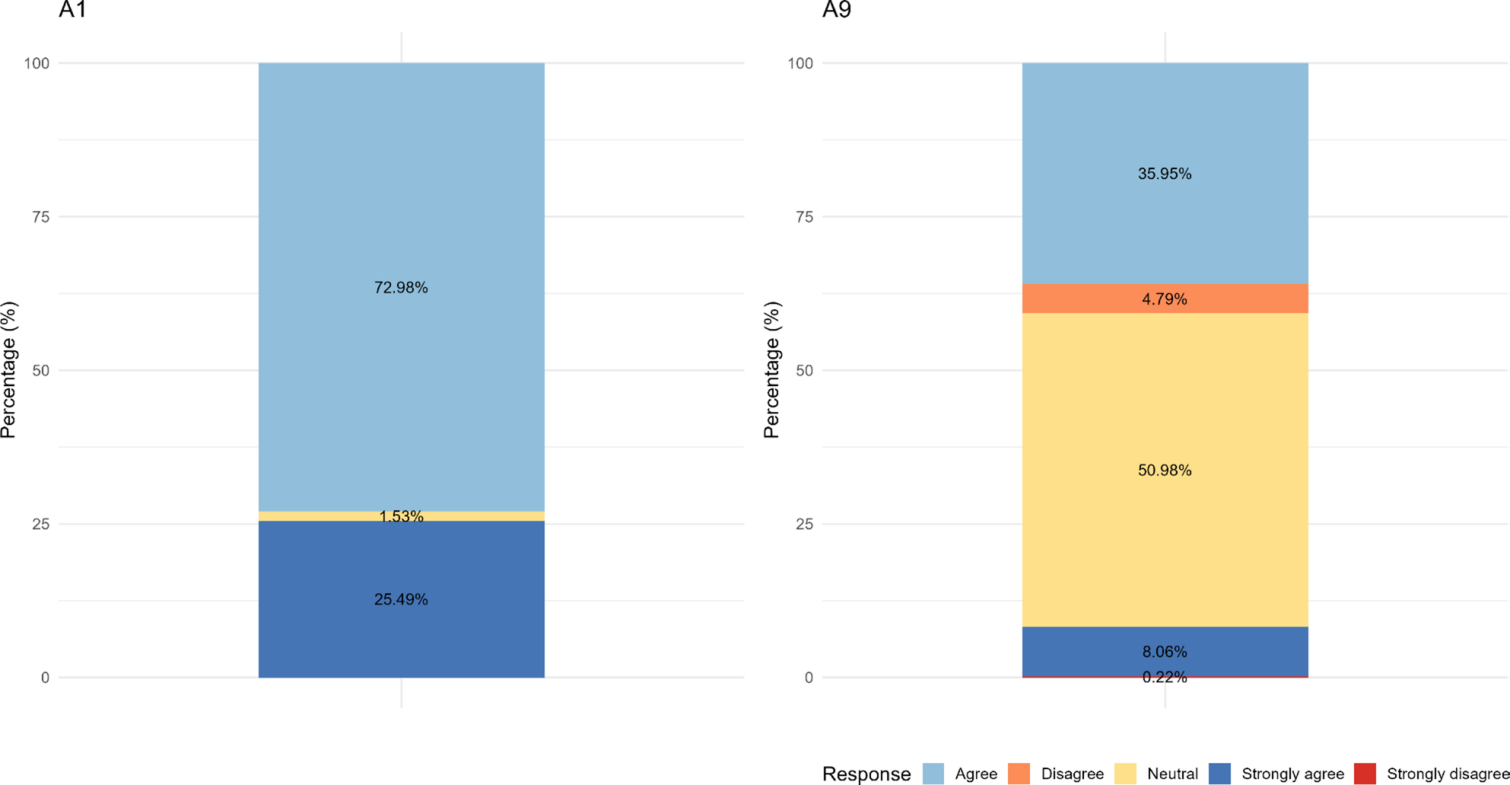
Key questions analysis of attitudes.

The strongest agreement was observed for the statement “I believe IRDs significantly affect patients’ quality of life,” with 98.47% of guardians agreeing or strongly agreeing. Conversely, the weakest positive responses appeared in “I feel optimistic about the future of family members with IRDs” (only 8.06% strongly agreed) and “I believe there is sufficient societal support for patients with IRDs” (9.37% strongly agreed), highlighting emotional uncertainty and perceived external support limitations (Table S3 and Fig. 2).

**Fig. 3:**
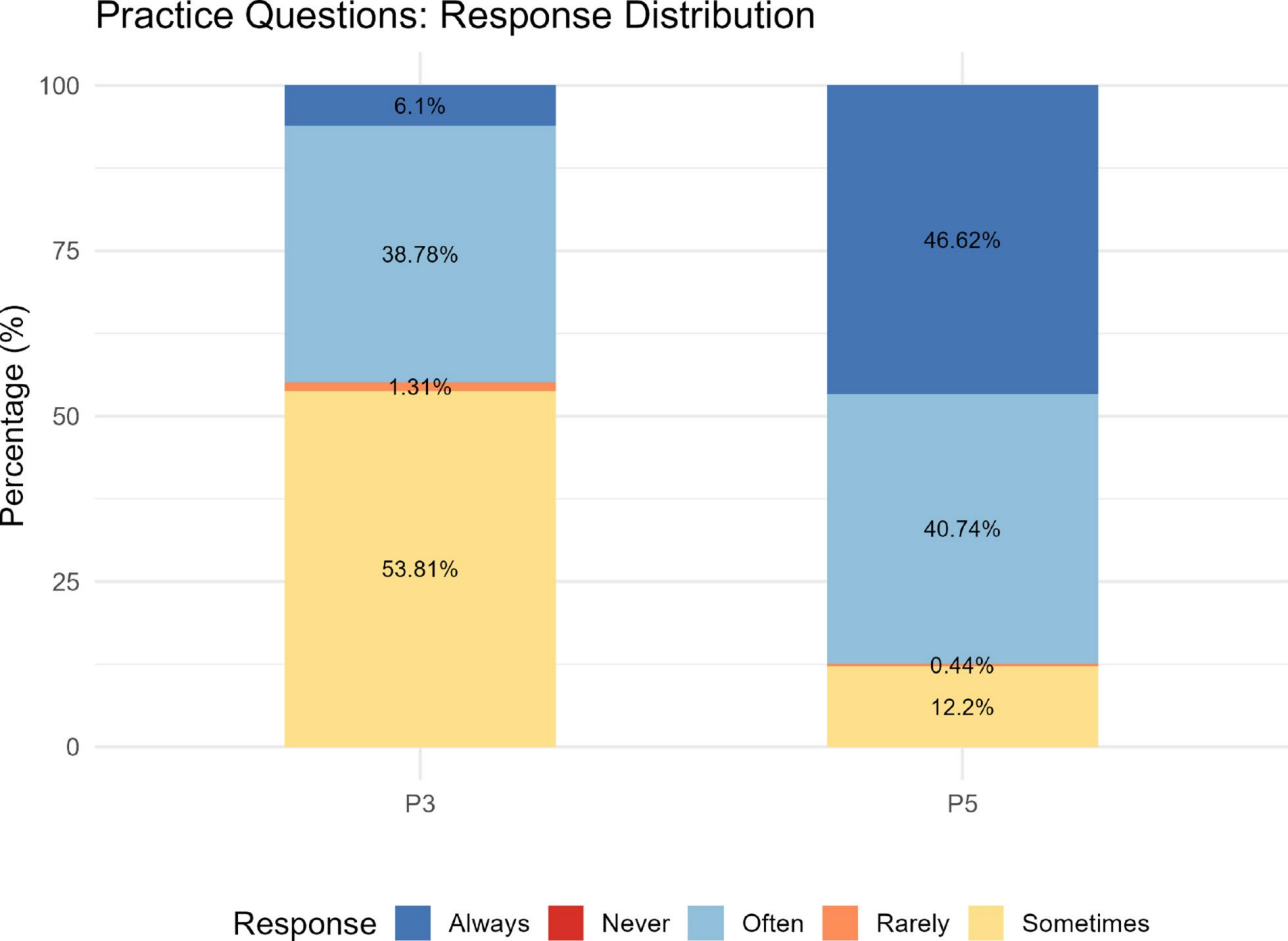
Key questions analysis of practices.

Guardians most frequently reported following medical advice, with 46.62% always complying with doctors’ recommendations. However, proactive practice was relatively limited; only 6.1% always sought assistive devices, and 6.54% consistently ensured nutritional support for affected children, indicating a gap between general compliance and resource-seeking practice (Table S4 and Fig. 3).

### Correlation analysis

In the correlation analysis, significant positive correlation was found between attitude and practice (*r* = 0.5741, *P* < 0.001). However, none of the correlations between knowledge and attitude as well as practice were statistically significant (Table 3).

**Table 3:** Correlation analysis.

	Knowledge	Attitude	Practice
Knowledge	1		
Attitude	0.0792 (*P* = 0.090)	1	
Practice	0.0026 (*P* = 0.956)	0.5741 (*P*<0.001)	1

### Path analysis

**Fig. 4:**
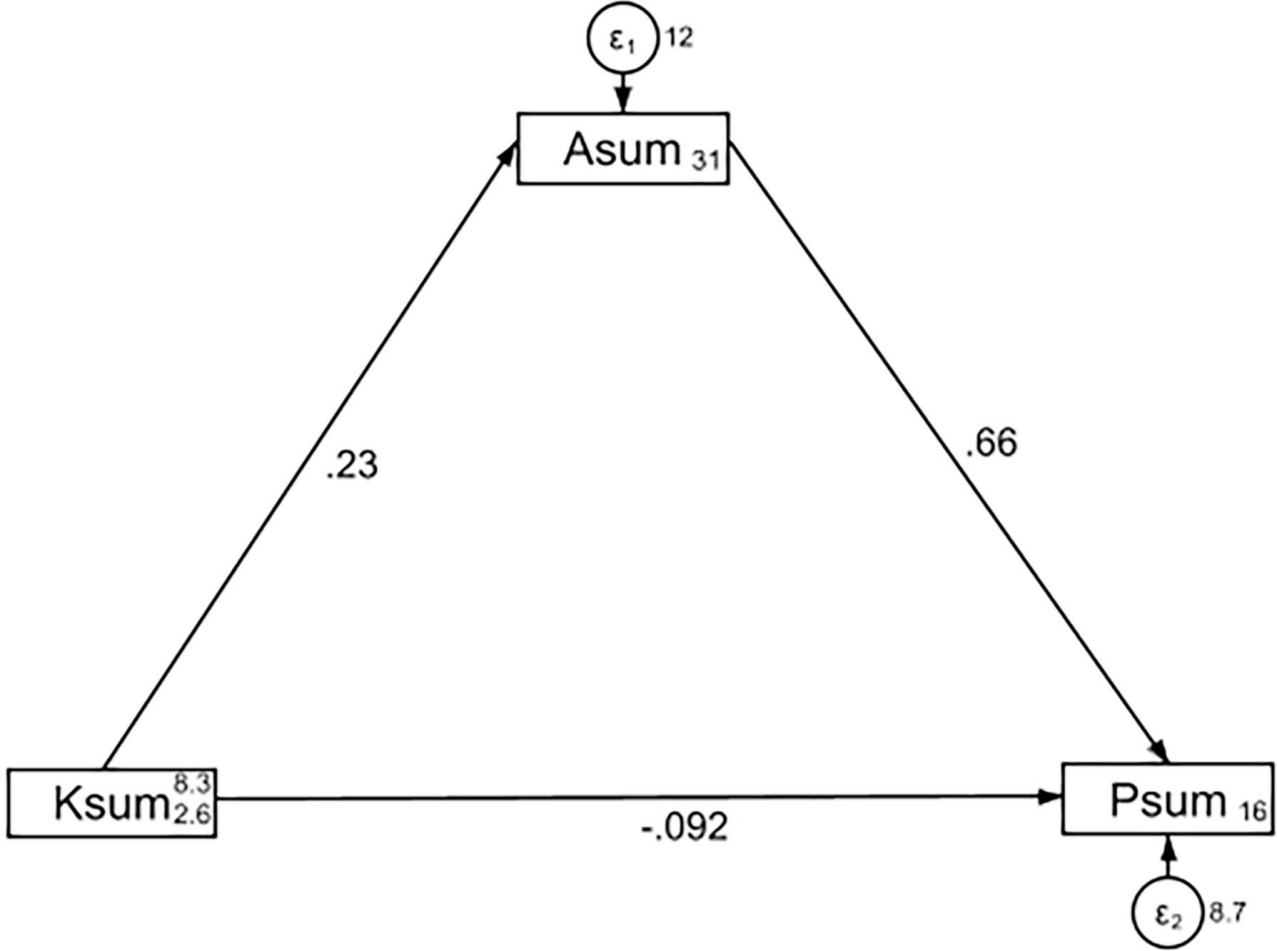
Path analysis model.

The path analysis showed that knowledge had a direct effect on attitude (β = 0.22, *P* = 0.023). Meanwhile, attitude had a direct effect on practice (β = 0.66, *P* < 0.001). Although the direct effect of knowledge on practice was not significant, it has an indirect effect on practice through attitude (β = 0.15, *P* = 0.024) (Table 4; Fig. 4).

**Table 4:** Analysis of direct and indirect effects.

Model paths		Total effects		Direct Effect		Indirect effect	
		β (95% CI)	*P*	β (95% CI)	*P*	β (95% CI)	*P*
Asum <-	Ksum	0.22(0.03,0.42)	0.023	0.22(0.03,0.42)	0.023		
Psum <-	Asum	0.66(0.58,0.74)	< 0.001	0.66(0.58,0.74)	< 0.001		
	Ksum	0.06(-0.15,0.27)	0.574	-0.09(-0.26,0.07)	0.287	0.15(0.01,0.28)	0.024

### Subgroup analysis

To further explore whether guardians’ KAP differed by disease type, two complementary subgroup analyses were conducted. First, considering the limited sample sizes of certain IRD subtypes, a binary subgroup comparison was performed between retinitis pigmentosa (RP) and non–retinitis pigmentosa (Non-RP) cases to reduce potential loss of statistical power. No significant differences were observed between the RP and Non-RP groups across knowledge (8.29 ± 1.66 vs. 8.37 ± 1.53, *P* = 0.437), attitude (33.25 ± 3.53 vs. 33.25 ± 3.49, *P* = 0.873), or practice scores (36.95 ± 3.61 vs. 36.89 ± 3.99, *P* = 0.798) (Table 5).

**Table 5:** Subgroup comparison between Retinitis Pigmentosa (RP) and Non-Retinitis Pigmentosa (Non-RP).

Mean ± SD	Retinitis Pigmentosa	Non-retinitis pigmentosa	*P*
K	8.291.66	8.371.53	0.437
A	33.253.53	33.253.49	0.873
P	36.953.61	36.893.99	0.798

Second, a detailed subgroup analysis was conducted across major IRD subtypes, including RP, Leber congenital amaurosis, Stargardt disease, choroideremia, and other IRDs. Consistent with the binary comparison, no statistically significant differences were observed in KAP scores across these IRD subtypes (Table 6). These findings suggest that guardians of children with different IRD diagnoses share broadly similar KAP, regardless of specific disease subtype.

**Table 6:** Subgroup comparison of KAP scores by IRD type.

Mean ± SD	Retinitis Pigmentosa	Leber Congenital Amaurosis	Stargardt disease	Choroideremia	Other	*P*
K	8.291.66	8.051.44	8.311.73	8.551.52	8.721.43	0.128
A	33.253.53	32.923.18	33.573.44	32.573.33	34.214.00	0.204
P	36.953.61	36.673.44	37.004.00	36.174.20	37.924.47	0.446

## Discussion

This study revealed that guardians of children diagnosed with IRDs exhibit limited knowledge, suboptimal attitudes, and relatively proactive practice. Path analysis confirms that knowledge does not directly influence practice but exerts an indirect effect through attitudes. These findings underscore the critical mediating role of parental attitudes between knowledge and caregiving practice. Therefore, in clinical and family caregiving settings, interventions should not only focus on enhancing guardians’ disease knowledge but also actively foster more positive and engaged attitudes to drive meaningful changes in practices.

This study highlighted several pressing challenges in the KAP of guardians whose children are affected by inherited retinal diseases. While most guardians demonstrated relatively active involvement in caregiving, particularly in following clinical recommendations and basic support tasks, a deeper analysis reveals a disconnect between what guardians know and how they think and act in response to the disease. Specifically, the non-significant Spearman correlation between knowledge and attitudes contrasts with the significant indirect effect observed in the path analysis. This discrepancy can be explained by the methodological differences between the two approaches. The Spearman correlation analyzes observed variables, which may not fully capture the latent constructs of knowledge and attitudes, while path analysis evaluates latent variables and considers indirect effects through attitudes, providing a more comprehensive understanding of the relationships among KAP^16^. A consistent pattern emerges in which knowledge alone does not appear sufficient to shape attitudes or drive change in practices. This aligns with findings in other fields of rare and genetic disorders, where even motivated caregivers often struggle to translate fragmented understanding into informed action^17,18^. The disjunction between cognitive awareness and practical engagement suggests that simply providing information may not be enough in the absence of supportive systems and emotionally resonant communication.

One of the clearest findings from this study is the strong relationship between attitude and practice. path analysis confirmed that attitude had a significant and direct influence on practice, indicating that what guardians believe—about the disease, its consequences, and the possibilities for management—plays a critical role in shaping what they do. This supports earlier research suggesting that emotional readiness and belief systems are often more influential than factual knowledge in determining health-related practice^19,20^. Notably, even when guardians lacked detailed understanding of genetics or treatment options, those who held more constructive or engaged views about the condition were more likely to take proactive steps, such as encouraging medical follow-up or seeking assistive resources. Interestingly, the significantly higher Attitude and Practice scores observed among “Other” guardians (primarily grandparents) compared to parents may be partially attributable to a potential social desirability bias among the elderly, who might tend to provide more favorable responses^21^. Additionally, while the difference was statistically significant, the small absolute score difference suggests limited practical relevance. These findings indicate that questionnaire data should be interpreted with caution, especially when comparing responses across different demographic groups.

Although most guardians correctly recognized that IRDs are hereditary (92.81%) and that genetic testing is necessary (84.53%), the overall level of knowledge in this cohort remained limited. Critical misconceptions were observed in understanding clinical heterogeneity—only 17.65% correctly acknowledged that the same gene can result in varied clinical manifestations—and in recognizing mitochondrial inheritance, with just 12.2% answering correctly. Uncertainty about treatment applicability was also widespread, as 70.59% were unsure whether options like gene therapy or supportive care were relevant. These findings indicate substantial gaps in basic genetic and clinical literacy. Such limitations are consistent with observations in other rare disease populations, where caregiver understanding often stems from anecdotal experience or fragmented communication, rather than structured education or counseling^22,23^. Additionally, the misperception that screening of unaffected family members is unnecessary—evidenced by 32.68% expressing uncertainty—may hinder timely identification and intervention. These patterns are consistent with research on childhood visual impairments, where studies have similarly documented that parental awareness of disease severity does not reliably predict understanding of treatment options or engagement in preventive practices^24,25^. Therefore, public education initiatives for IRDs should place emphasis on explaining concepts such as “why siblings with the same gene mutation can have different clinical outcomes.” This approach, akin to strategies in hereditary cancer awareness^26^, helps enhance public understanding of genetic variability and underscores the importance of early diagnosis and intervention.

Parental attitudes reflected a similar ambivalence. Although most participants expressed concern about the quality of life impact associated with IRDs, relatively few conveyed optimism about treatment prospects or the future of their children. A considerable proportion remained neutral on key statements, such as the benefits of genetic counseling or the value of emotional support. This pattern of guarded or indifferent attitudes has been observed in other studies of families coping with progressive visual impairment, where uncertainty about prognosis and lack of visible therapeutic outcomes may contribute to emotional disengagement or resignation^27^. In this study, more favorable attitudes were associated with factors such as urban residence, higher educational attainment, and prior exposure to disease-related education, suggesting that structural access to resources and environments that support active learning can make a meaningful difference in caregiver outlook. In contrast, the subgroup analyses showed that guardians’ KAP did not differ significantly across major IRD subtypes, RP and Non-RP groups. This finding suggests that, despite clinical and genetic heterogeneity among IRDs, caregivers may share broadly similar experiences, concerns, and informational needs when managing these conditions. The absence of subtype-specific differences in KAP may reflect common challenges faced by families of children with progressive retinal diseases, such as uncertainty regarding prognosis, limited access to specialized genetic counseling, and a lack of tailored educational resources^28^. From a practical perspective, these results indicate that general educational and support interventions targeting guardians of children with IRDs may be broadly applicable across different disease types, rather than requiring highly subtype-specific approaches^29^.

In terms of practice, while most guardians reported adherence to physician recommendations and regular involvement in care, engagement in more complex or forward-looking practice—such as participation in clinical research or discussions about the disease within the family—was less consistent. This mirrors findings from other studies where logistical constraints, cultural stigma, and lack of tailored support often inhibit deeper forms of engagement^30^. For example, discussing genetic risk within families or advocating for specialized services may require not only knowledge but also confidence, psychological support, and a broader understanding of long-term management.

The demographic analysis further reinforces the influence of socioeconomic context on caregiver practice. Guardians with higher education or household income tended to show more positive attitudes and practice, while those from rural areas or with limited educational backgrounds reported lower scores across these dimensions. These findings reflect broader trends in health inequality, where access to quality information and services remains unevenly distributed. Similar disparities have been reported in studies from other countries, suggesting that the barriers observed here are not unique but part of a wider pattern of systemic limitation^31^.

Based on these findings, several recommendations can be made. Health education strategies should extend beyond clinical settings and include community-based outreach, particularly in underserved areas. Printed materials, short videos, and locally adapted digital tools could be developed in collaboration with patient organizations and medical institutions to explain IRDs in simple, accurate, and engaging ways. These resources should be designed not only to convey information but also to correct misconceptions and encourage dialogue within families. In clinical practice, the integration of routine genetic counseling into ophthalmology services should be prioritized. Given that many guardians had limited understanding of inheritance and prevention strategies, accessible and repeated counseling sessions may help bridge the gap between diagnosis and long-term management. Providers should be trained to deliver this information in a manner that is sensitive to emotional readiness and tailored to different levels of education^32–34^.

This study has several limitations that should be considered when interpreting the findings. First, the use of a self-developed questionnaire, although tailored to the study objectives, may limit comparability with other research and introduce measurement bias. Additionally, all KAP measures were self-reported, which may introduce recall bias and social desirability bias. Second, although this study, based on the KAP theory, identified direct and indirect associations among KAP components, wherein knowledge is viewed as a precursor to attitude formation, and attitude subsequently influences practical behavior^35^, its cross-sectional design precludes the establishment of causal relationships among these variables. Additionally, the lack of longitudinal follow-up limits our ability to track changes in KAP over time or to evaluate the effects of interventions. Third, the questionnaire focused on basic knowledge and general attitudes, not capturing deeper clinical understanding or actual caregiving behaviors, which may limit the interpretability of the results. In addition, questionnaire did not include items on future planning (e.g., education, career considerations) as it focused on immediate caregiving practices and knowledge. Future studies may consider including such items to assess guardians’ preparedness for long-term care. Finally, the inclusion of highly specialized and challenging items contributed to the lower overall mean score, which may be misinterpreted. Future studies could better balance basic and advanced items for a more comprehensive assessment on knowledge. These advancements in treatment underscore the need for better education and information dissemination to ensure that guardians are aware of the potential treatment options available. As new therapies emerge, it will be crucial to address these knowledge gaps in future educational programs for caregivers of children with IRDs.

Looking forward, ongoing advances in gene therapy and other therapeutic interventions offer hope for families affected by IRDs. For instance, phase 1/2 trials of botaretigene sparoparvovec for X-linked retinitis pigmentosa have demonstrated safety and preliminary efficacy, with phase 3 trials currently underway^36^. As these emerging treatments progress toward clinical application, educational interventions should incorporate information about therapeutic developments to help guardians make informed decisions and maintain realistic yet optimistic expectations for their children’s care.

In conclusion, guardians of pediatric patients diagnosed with IRDs demonstrated insufficient knowledge and suboptimal attitudes, despite engaging in relatively proactive health-related practice. These findings highlight the need for enhanced disease-related education on clinical variability to improve parental knowledge, foster more positive attitudes, and promote proactive health practice.

## Supplementary Information

Below is the link to the electronic supplementary material.

Supplementary Material 1

## Data Availability

All data generated or analysed during this study are included in this published article.
