# Supplementary material for: Knowledge, attitudes, and practices among guardians toward inherited retinal diseases: a structural equation modeling analysis
Source: Sci Rep. 2026 May 21;16:23229. doi: 10.1038/s41598-026-54188-7 (PMC13402300; doi:10.1038/s41598-026-54188-7)
Supplement: Supplementary file 1 — Supplementary Material 1 [file 41598_2026_54188_MOESM1_ESM.docx]

**Table S1. Fit indices of confirmatory factor analysis.**

| Indicators | Reference | Actual |
| --- | --- | --- |
| CMIN/DF | 1-3: Excellent, 3-5: Good | 1.460 |
| RMSEA | <0.08: Good | 0.032 |
| IFI | >0.8: Good | 0.909 |
| TLI | >0.8: Good | 0.894 |
| CFI | >0.8: Good | 0.907 |

**Table S2. Distribution of knowledge dimension responses**

| **Knowledge** | **N(%)** | | | | |
| --- | --- | --- | --- | --- | --- |
|  | **True** | | **False** | **Not sure** | |
| 1. IRDs may be passed down through genes. | 426(92.81) | | 8(1.74) | 25(5.45) | |
| 1. Patients with IRDs need to undergo genetic testing (genetic diagnosis). | 388(84.53) | | 12(2.61) | 59(12.85) | |
| 1. The same pathogenic gene can present with significantly different clinical manifestations in IRDs. | 81(17.65) | | 71(15.47) | 307(66.88) | |
| 1. It is unnecessary for the parents, siblings, or children of patients with IRDs to be screened for pathogenic genes. | 72(15.69) | | 237(51.63) | 150(32.68) | |
| 1. IRDs can be prevented. | 389(84.75) | | 8(1.74) | 62(13.51) | |
| 1. Drug therapy, gene therapy, surgical treatment, and supportive care are all treatment methods for IRDs. | 90(19.61) | | 45(9.8) | 324(70.59) | |
| 1. Early diagnosis may influence the progression of IRDs. | 321(69.93) | | 19(4.14) | 119(25.93) | |
| 8. The most common symptom of IRDs is: |  | |  |  | |
| A. Night blindness | | 216(47.06) | | |  |
| B. Myopia | | 46(10.02) | | |  |
| C. Astigmatism | | 23(5.01) | | |  |
| D. Cataract | | 1(0.22) | | |  |
| E. Not sure | | 173(37.69) | | |  |
| 9. Which of the following is *not* a treatment method for IRDs? | |  | | |  |
| A. Drug therapy | | 3(0.65) | | |  |
| B. Gene therapy | | 6(1.31) | | |  |
| C. Nutritional support | | 299(65.14) | | |  |
| D. Laser surgery | | 84(18.3) | | |  |
| E. Not sure | | 67(14.6) | | |  |
| 10. The diagnosis of IRDs mainly relies on: | |  | | |  |
| A. Patient self-report | | 2(0.44) | | |  |
| B. Family history | | / | | |  |
| C. Genetic testing | | 280(61) | | |  |
| D. Vision test | | 17(3.7) | | |  |
| E. Not sure | | 160(34.86) | | |  |
| 11. Which of the following is a preventive measure for IRDs? | |  | | |  |
| A. Avoiding sun exposure | | / | | |  |
| B. Regular eye examinations | | 22(4.79) | | |  |
| C. Avoiding consanguineous marriage | | 302(65.8) | | |  |
| D. All of the above | | 54(11.76) | | |  |
| E. Not sure | | 81(17.65) | | |  |
| 12. Common onset times of IRDs include (select all that apply) | |  | | |  |
| A. Childhood | | 456(99.35) | | |  |
| B. Adolescence | | 446(97.17) | | |  |
| C. Adulthood | | 304(66.23) | | |  |
| D. Old age | | 19(4.14) | | |  |
| E. Not sure | | / | | |  |
| 13. What are the possible inheritance patterns of IRDs? (select all that apply) | |  | | |  |
| A. Autosomal dominant inheritance | | 453(98.69) | | |  |
| B. Autosomal recessive inheritance | | 452(98.47) | | |  |
| C. Sex-linked inheritance | | 451(98.26) | | |  |
| D. Mitochondrial inheritance | | 56(12.2) | | |  |
| E. Not sure | | 2(0.44) | | |  |
| 14. Family members of patients with IRDs may need: (select all that apply) | |  | | |  |
| A. Genetic counseling | | 456(99.35) | | |  |
| B. Genetic testing | | 451(98.26) | | |  |
| C. Regular eye examinations | | 456(99.35) | | |  |
| D. Psychological support | | 191(41.61) | | |  |
| E. Not sure | | / | | |  |

**Table S3. Distribution of attitude dimension responses**

| **Attitude** | **Strongly agree** | **Agree** | **Neutral** | **Disagree** | **Strongly disagree** |
| --- | --- | --- | --- | --- | --- |
| 1. I believe IRDs significantly affect patients’ quality of life. | 117(25.49) | 335(72.98) | 7(1.53) | / | / |
| 2. I believe gene therapy can effectively treat IRDs. | 45(9.8) | 339(73.86) | 70(15.25) | 5(1.09) | / |
| 3. I believe providing psychological and emotional support to patients with IRDs is very necessary. | 36(7.84) | 178(38.78) | 232(50.54) | 13(2.83) | / |
| 4. I believe parents, siblings, or children of individuals diagnosed with IRDs should undergo genetic screening and actively participate in pedigree analysis. | 36(7.84) | 171(37.25) | 235(51.2) | 17(3.7) | / |
| 5. I believe proactive genetic counseling and prenatal diagnosis can reduce the incidence of IRDs to some extent. | 55(11.98) | 181(39.43) | 209(45.53) | 14(3.05) | / |
| 6. I have a positive attitude toward the treatment of IRDs. | 74(16.12) | 212(46.19) | 161(35.08) | 12(2.61) | / |
| 7. I believe genetic counseling is important for the families of patients with IRDs. | 56(12.2) | 240(52.29) | 155(33.77) | 8(1.74) | / |
| 9. I feel optimistic about the future of family members with IRDs. | 37(8.06) | 165(35.95) | 234(50.98) | 22(4.79) | 1(0.22) |
| 10. I believe there is sufficient societal support for patients with IRDs. | 43(9.37) | 165(35.95) | 228(49.67) | 23(5.01) | / |

**Table S4. Distribution of practice dimension responses**

| **Practice** | **Always** | **Often** | **Sometimes** | **Rarely** | **Never** |
| --- | --- | --- | --- | --- | --- |
| 1. I actively help family members with IRDs find and access medical resources. | 58(12.64) | 311(67.76) | 89(19.39) | 1(0.22) | / |
| 1. Family members actively undergo genetic testing for fundus-related diseases before childbirth. | 48(10.46) | 263(57.3) | 144(31.37) | 4(0.87) | / |
| 1. I actively seek and try various assistive devices to help improve the patient’s vision. | 28(6.1) | 178(38.78) | 247(53.81) | 6(1.31) | / |
| 1. I actively monitor regular visual function evaluations of family members with IRDs. | 48(10.46) | 183(39.87) | 218(47.49) | 10(2.18) | / |
| 1. I follow the doctor’s recommendations for treating the patient. | 214(46.62) | 187(40.74) | 56(12.2) | 2(0.44) | / |
| 1. I encourage family members with IRDs to actively participate in clinical trials for gene therapy in China. | 45(9.8) | 231(50.33) | 174(37.91) | 9(1.96) | / |
| 1. I ensure that family members with IRDs receive necessary nutritional support. | 30(6.54) | 190(41.39) | 220(47.93) | 19(4.14) | / |
| 1. I discuss the disease with the patient to enhance their understanding of IRDs. | 36(7.84) | 206(44.88) | 203(44.23) | 14(3.05) | / |
| 1. I encourage and support family members with IRDs to participate in physical and recreational activities suitable for their visual condition. | 36(7.84) | 191(41.61) | 216(47.06) | 16(3.49) | / |
| 1. I learn about IRDs together with other family members. | 33(7.19) | 193(42.05) | 209(45.53) | 24(5.23) | / |
